# Isolated Paramedian Pontine Reticular Formation (PPRF) syndrome in AQP4 antibody–positive neuromyelitis optica spectrum disorder: a case report

**DOI:** 10.1186/s12883-026-04670-6

**Published:** 2026-01-29

**Authors:** Manami Terahara, Takahiro Hobara, Yujiro Higuchi, Shota Hirakata, Yutaka Noguchi, Satoshi Nozuma, Hiroshi Takashima

**Affiliations:** Department of Neurology and Geriatrics, Kagoshima University Graduate School of Medical and Dental Sciences, 8-35-1 Sakuragaoka, Kagoshima City, 890-8520 Kagoshima Japan

**Keywords:** Aquaporin-4 (AQP4), Neuromyelitis optica spectrum disorder (NMOSD), Horizontal gaze palsy, Paramedian pontine reticular formation (PPRF) syndrome

## Abstract

**Background:**

Paramedian pontine reticular formation (PPRF) syndrome is characterized by horizontal gaze palsy due to lesions in the PPRF or the abducens nucleus. It is relatively rare and is typically associated with medial longitudinal fasciculus (MLF) syndrome.

**Case presentation:**

A 53-year-old Japanese woman presented with right lateral gaze palsy and facial nerve palsy, with preserved convergence and left lateral gaze. Neurological evaluation confirmed PPRF syndrome and facial nerve palsy, with no involvement of the MLF. Brain MRI showed a lesion in the right dorsal pons, affecting the PPRF, abducens nucleus, and facial nerve. Serum testing revealed positive antinuclear and anti-AQP4 antibodies. The patient responded well to immunotherapy, with substantial clinical and radiological improvements. She was ultimately diagnosed with AQP4 antibody-positive NMOSD.

**Conclusion:**

This is the first documented case of isolated PPRF syndrome in AQP4 antibody–positive NMOSD. The findings expand the known phenotypic spectrum of NMOSD and highlight the importance of considering NMOSD, with targeted AQP4 antibody testing, in patients presenting with isolated PPRF syndrome.

## Background

Paramedian pontine reticular formation (PPRF) syndrome is defined by horizontal gaze palsy resulting from lesions in the PPRF or the abducens nucleus. The PPRF plays a central role in coordinating horizontal eye movements by transmitting signals from the frontal eye field to the abducens nucleus, which in turn facilitates conjugate lateral gaze. In contrast, medial longitudinal fasciculus (MLF) syndrome, also referred to as internuclear ophthalmoplegia (INO), is characterized by unilateral impairment of ocular adduction due to lesions in the paramedian tegmental region. MLF syndrome is more frequently observed and associated with conditions like multiple sclerosis (MS) and cerebrovascular diseases [1, 2]. In comparison, PPRF syndrome is rare and often underrecognized in clinical practice.

PPRF syndrome is frequently seen alongside MLF involvement, leading to one-and-a-half syndrome—a condition characterized by unilateral horizontal gaze palsy and contralateral adduction impairment [3]. Although numerous cases of MLF syndrome and one-and-a-half syndrome have been documented, isolated PPRF syndrome without MLF involvement is exceptionally rare, particularly in neuromyelitis optica spectrum disorder (NMOSD).

To the best of our knowledge, this is the first reported case of lateral gaze palsy caused by PPRF syndrome without MLF involvement, accompanied by ipsilateral abducens nucleus and facial nerve palsy, in a patient with aquaporin-4 (AQP4) antibody–positive NMOSD.

## Case presentation

The patient was a 53-year-old Japanese woman previously diagnosed with systemic lupus erythematosus at the age of 37 years. Reasonable disease control had been achieved with oral prednisolone, hydroxychloroquine, and belimumab.

**Fig. 1:**
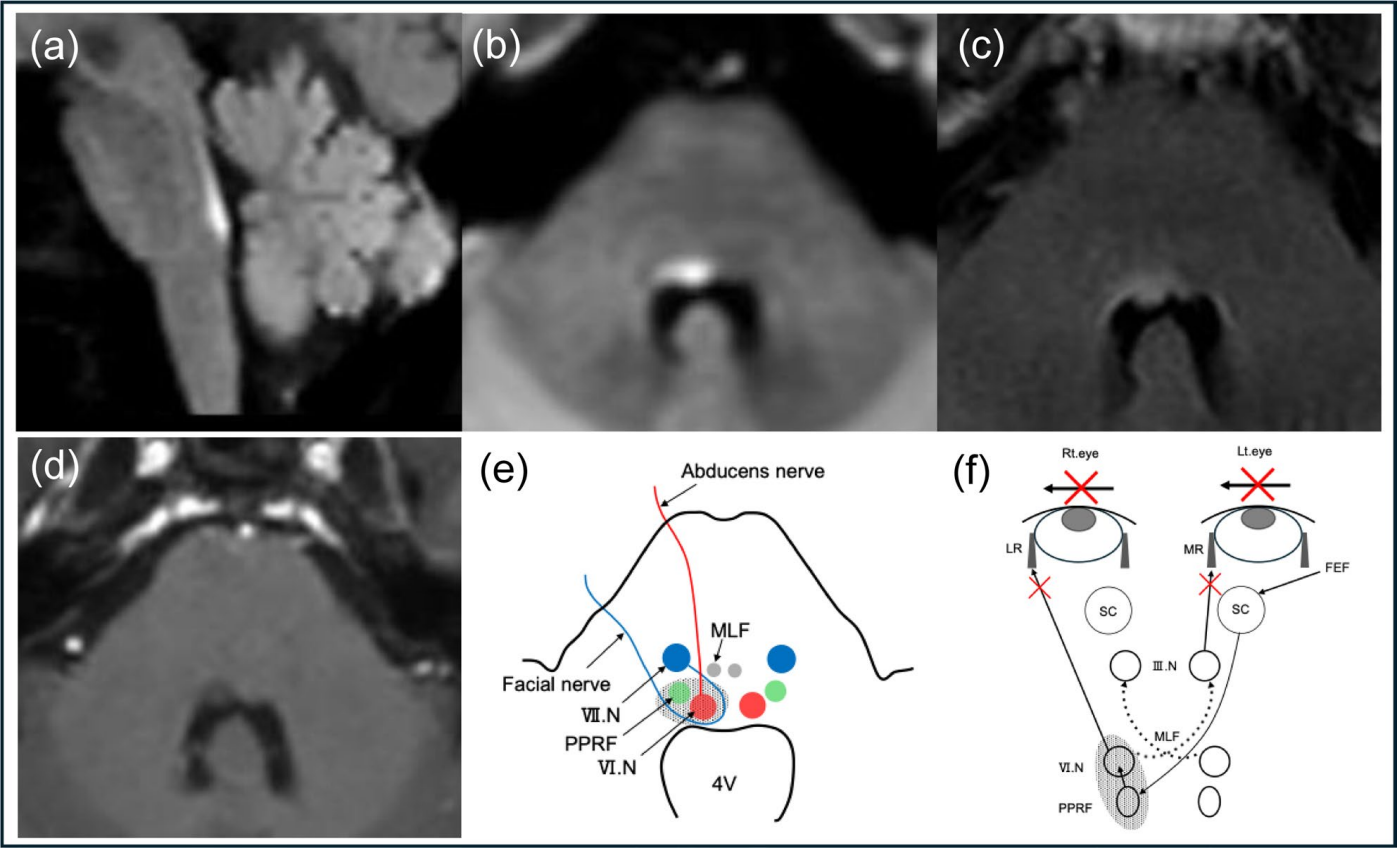
Brain MRI on day 9 after onset. DWI (**a**–**b**) demonstrates restricted diffusion along the dorsal pons and around the fourth ventricle, with FLAIR (**c**) sequences showing corresponding high signal intensity. T1 contrast-enhanced images reveal no enhancement in the lesion (**d**). Schematic of the horizontal section: the lesion involves the right PPRF, abducens nucleus, and infranuclear facial nerve, sparing the MLF (**e**). Schematic of ocular movements: the lesion in the right PPRF and abducens nucleus results in right lateral rectus muscle and left medial rectus muscle palsies due to contralateral oculomotor nucleus involvement, ultimately leading to lateral gaze palsy (**f**). MRI, magnetic resonance imaging; DWI, diffusion-weighted imaging; FLAIR, fluid-attenuated inversion recovery; Ⅲ.N, oculomotor nucleus; Ⅵ.N, abducens nucleus; Ⅶ.N, facial nerve nucleus; MLF, medial longitudinal fasciculus; PPRF, paramedian pontine reticular formation; 4V, fourth ventricle; FEF, frontal eye field; SC, superior colliculus; LR, lateral rectus muscle; MR, medial rectus muscle

**Fig. 2:**
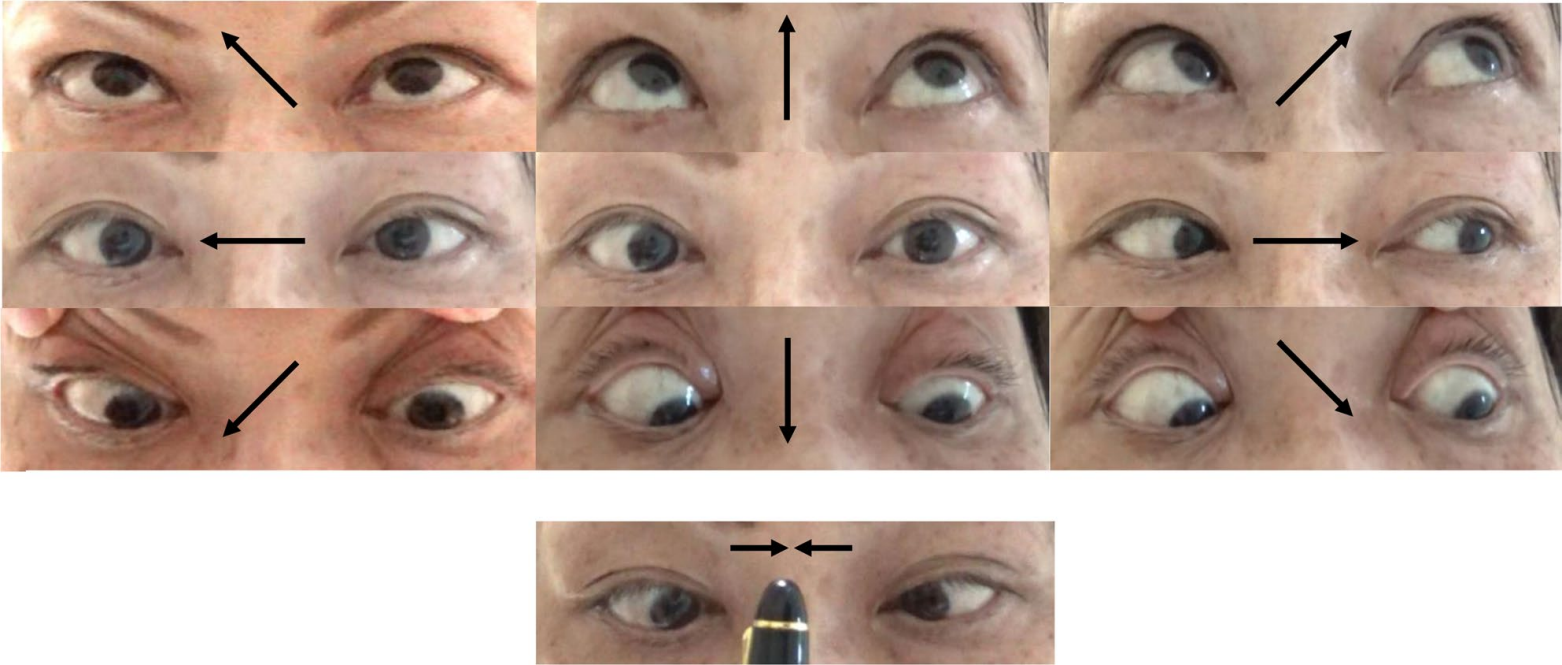
Images of the ocular movements: right lateral gaze palsy is present, whereas left lateral gaze, vertical ocular movements, and convergence are fully intact. The right eye is adducted, whereas the left eye is normal in the primary position. Arrows indicate the direction of the attempted gaze

At the age of 53, the patient initially experienced diplopia upon awakening with limited ocular movement in the right eye, incomplete eyelid closure on the right side, and drooling from the right corner of the mouth when drinking. Nausea developed on day 5 after onset, and T2-weighted magnetic resonance imaging (MRI) carried out on day 9 after onset revealed an elongated, high-signal lesion along the right dorsal part of the pons (Fig. 1a–d). Furthermore, neurological examination carried out on day 25 after onset revealed the right eye to be in an adducted position in the primary position, with complete abduction palsy observed during voluntary gaze and vestibulo-ocular reflex. The left eye was normal in the primary position but exhibited adduction paresis. No ptosis was detected, and the left lateral and vertical gazes were intact. Moreover, convergence was fully intact, indicating normal bilateral oculomotor nerve function (Fig. 2). Horizontal nystagmus was observed in the left eye during left gaze, but no nystagmus was detected in the right eye. Additionally, peripheral facial nerve palsy on the right side was observed. There were no signs of optic neuritis, dizziness, dysarthria, dysphagia, hiccups, limb weakness, sensory disturbance, cerebellar ataxia, or pyramidal tract. Blood tests at the time of the first admission (on day 25 after onset) revealed serum positivity for AQP4 antibodies, with an ELISA antibody level of 10.6 U/mL, which was further confirmed by a positive cell-based assay. Antinuclear antibodies were positive, whereas anti–double-stranded DNA antibody test was negative, and complement components C3 and C4 were within normal limits, arguing against active SLE or neuropsychiatric SLE involvement.

Cerebrospinal fluid exhibited normal cell count as well as protein and IgG levels, with positive oligoclonal bands (OCBs) and elevated interleukin-6 level (13.5 pg/mL). Spinal MRI revealed no significant abnormalities.

**Fig. 3:**
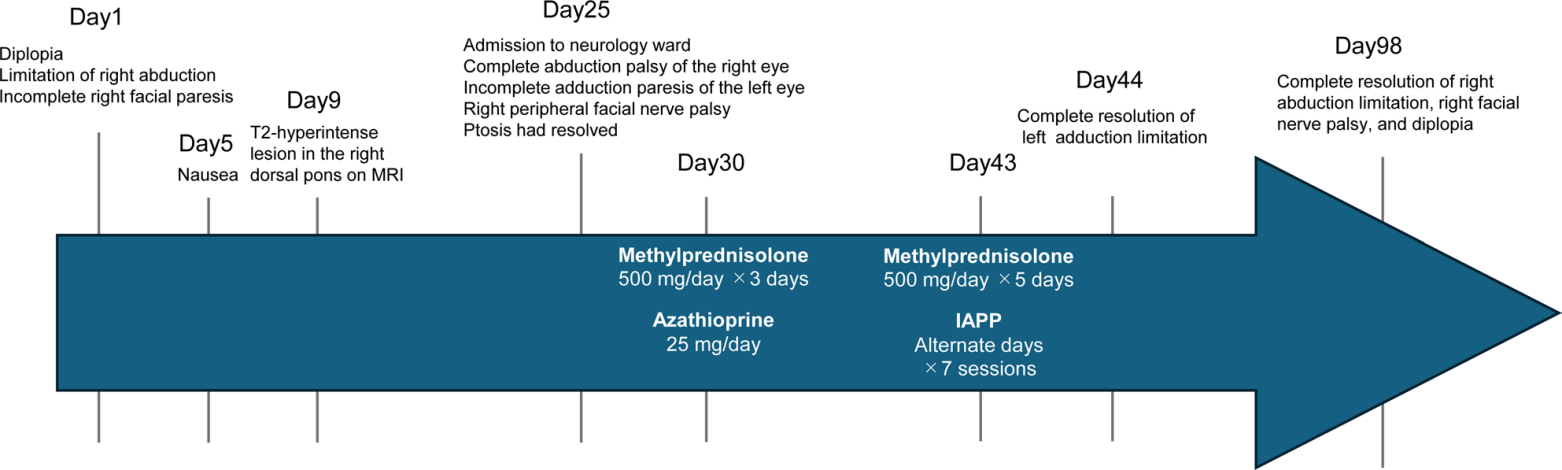
Clinical timeline of symptoms, imaging, and treatment. Day 1: the patient developed diplopia, limitation of right abduction, and incomplete right facial paresis. Day 9: brain MRI revealed a T2-hyperintense lesion in the right dorsal pons. Day 25: the patient was admitted with complete right abduction palsy, incomplete left adduction paresis, and right peripheral facial nerve palsy; ptosis had resolved. Immunotherapy was initiated on day 30 (methylprednisolone and azathioprine), followed by a second course of steroids and immunoadsorption plasmapheresis (IAPP) on day 43. Left adduction limitation resolved by day 44, and right abduction limitation, facial nerve palsy, and diplopia completely resolved by day 98

On day 30, intravenous methylprednisolone (500 mg/day for 3 days) and azathioprine (25 mg/day) were initiated in accordance with our institutional practice. The left-eye adduction deficit improved in response to treatment, but right-eye abduction palsy and right peripheral facial nerve palsy remained unchanged. On day 43, immunoadsorption plasmapheresis was performed and a second course of intravenous methylprednisolone was administered. By day 44 after onset, the adduction paresis had improved in the left eye, and by day 98 lateral gaze palsy, diplopia, and facial nerve palsy had resolved. Follow-up brain MRI revealed attenuation of the previous brainstem lesions on T2-weighted images, and no new lesions were observed. Based on the presence of AQP4 antibodies and the favorable response to immunotherapy, a diagnosis of NMOSD was made. A concise clinical timeline summarizing symptom onset, investigations, treatments, and recovery milestones is provided in Fig. 3.

## Discussion

Neural circuit for horizontal gaze originates from the frontal eye field in the cerebral cortex and transmits the PPRF, subsequently reaching the ipsilateral abducens nucleus. Signals are relayed from the abducens nucleus to the ipsilateral lateral rectus muscle, and medial rectus muscle of the opposite eye via the contralateral MLF and oculomotor nerve, enabling lateral gaze [4]. PPRF syndrome develops when a lesion in either the PPRF region or the abducens nucleus (excluding infranuclear) causes ipsilateral abduction and contralateral adduction deficits, leading to lateral gaze palsy toward the affected side (Fig. 1e, f) [4, 5]. These structures are anatomically close and often affected simultaneously. Owing to its extended anatomical course, the MLF is more susceptible to damage. Thus, isolated PPRF syndrome is exceedingly rare.

The persistent right lateral gaze palsy observed in the present case along with intact convergence that ruled out oculomotor nerve palsy as the cause of the adduction deficit in the left eye, suggested that the right lateral gaze palsy arose due to dysfunction in the PPRF and the right abducens nucleus. Despite nystagmus in the contralateral eye, the absence of adduction palsy in the right eye, and paralytic pontine exotropia in the left eye excluded both MLF syndrome and one-and-a-half syndrome. The MRI findings suggested that the lesion was confined to the dorsal aspect of the pons, involving the PPRF and adjacent structures—the abducens nucleus and facial nerve—while preserving the MLF pathway.

Patients with NMOSD present with various brainstem syndromes, while other inflammatory demyelinating disorders, such as MS and myelin oligodendrocyte glycoprotein antibody-associated disease (MOGAD), also produce diverse arrays of brainstem lesions. However, these conditions are generally distinguishable based on their characteristic clinical and radiological features. In NMOSD, lesions commonly affect the medulla oblongata, particularly for the area postrema [6, 7]. By contrast, MS typically features smaller, ovoid lesions in the pons or midbrain and exhibits a higher incidence of INO, diplopia, and trigeminal neuralgia [7]. Classic area postrema lesions, accompanied by severe nausea, vomiting, hiccups, or central respiratory dysfunction, are more characteristic of NMOSD than of MS [6, 7]. MOGAD can also present with extensive brainstem lesions—occasionally involving the central cerebellar peduncle or dorsal brainstem—and may partially overlap with both MS and NMOSD in clinical manifestations and imaging findings [8]. 

The prevalence of brainstem symptoms varies among these conditions. Approximately 16–22% of MS patients present with brainstem or cerebellar symptoms at disease onset, and more than half develop brainstem involvement during the disease course [9, 10]. By contrast, 7–10% of NMOSD patients present with isolated brainstem symptoms at disease onset, and overall brainstem involvement is reported in approximately 22–47% of patients, which is lower than that observed in MS [6, 11–15]. Brainstem involvement has also been reported in approximately 30% of patients with MOGAD [8, 13]. Thus, although MS often begins with optic neuritis or sensory deficits and subsequently involves the brainstem, NMOSD can present with brainstem manifestations as an initial clinical event.

Among the various ocular motor abnormalities in inflammatory demyelinating disorders, INO is often observed in MS [2, 16], but is relatively uncommon in NMOSD [17]. There have been a few reports of INO in NMOSD, including presentations such as one-and-a-half syndrome and wall-eyed bilateral internuclear ophthalmoplegia (WEBINO) syndrome [18, 19]. When INO or PPRF syndrome occurs in NMOSD without concurrent optic nerve or spinal cord involvement, there is a risk that NMOSD may go unrecognized. CSF OCBs are frequently positive in multiple sclerosis and serve as a useful discriminative biomarker. Although OCBs were positive in this case, they can occur in a minority of AQP4 antibody–positive NMOSD patients [20]. Therefore, differentiation between MS and NMOSD should not rely on OCBs alone and should include targeted AQP4 antibody testing. In our case, the isolated focal pontine syndrome in an AQP4-positive NMOSD patient underscores that NMOSD can manifest as isolated brainstem attacks, emphasizing the importance of prompt antibody testing. Considering the recent development of therapies for NMOSD targeting complement proteins C5, IL-6, and CD19 [21], misdiagnosis could delay the initiation of appropriate treatment. Thus, NMOSD should remain a diagnostic consideration even in atypical presentations such as isolated INO or PPRF syndrome.

## Conclusion

To the best of our knowledge, this is the first report of PPRF syndrome without MLF syndrome in a patient with AQP4 antibody–positive NMOSD. The absence of MLF involvement indicated that this is not a case of one-and-a-half syndrome. This case expands the known phenotypic spectrum of NMOSD and provides insights into the ocular motor dysfunction in PPRF syndrome. The presence of isolated PPRF syndrome supports the inclusion of NMOSD in the differential diagnosis alongside MS and highlights the value of targeted AQP4 antibody testing for accurate diagnosis.

## Data Availability

The data that support the findings of this study are available. Requests should be directed to the corresponding author.

## Abbreviations

PPRF: Paramedian pontine reticular formation
MLF: Medial longitudinal fasciculus
INO: Internuclear ophthalmoplegia
MS: Multiple sclerosis
NMOSD: Neuromyelitis optica spectrum disorder
AQP4: Aquaporin-4
MRI: Magnetic resonance imaging
OCBs: Oligoclonal bands
MOGAD: Myelin oligodendrocyte glycoprotein antibody-associated disease
WEBINO: Wall-eyed bilateral internuclear ophthalmoplegia
